# Carboxypeptidase 4 gene variants and early-onset intermediate-to-high risk prostate cancer

**DOI:** 10.1186/1471-2407-9-69

**Published:** 2009-02-26

**Authors:** Phillip L Ross, Iona Cheng, Xin Liu, Mine S Cicek, Peter R Carroll, Graham Casey, John S Witte

**Affiliations:** 1 Department of Urology, University of California San Francisco, San Francisco, CA, USA; 2 Department of Epidemiology and Biostatistics and Institute for Human Genetics, University of California San Francisco, San Francisco, CA, USA; 3 Division of Experimental Pathology and Laboratory Medicine, Mayo Clinic, Rochester, MN, USA; 4 Department of Preventive Medicine, University of Southern California, Los Angeles, CA, USA

## Abstract

**Background:**

Carboxypeptidase 4 *(CPA4) *is a zinc-dependent metallocarboxypeptidase on chromosome 7q32 in a region linked to prostate cancer aggressiveness. CPA4 is involved in the histone hyperacetylation pathway and may modulate the function of peptides that affect the growth and regulation of prostate epithelial cells. We examined the association between genetic variation in *CPA4 *and intermediate-to-high risk prostate cancer.

**Methods:**

We studied 1012 men (506 cases and 506 controls) from Cleveland, Ohio. All cases had Gleason ≥ 7, clinical stage ≥ T2c, or PSA ≥ 10 ng/mL at diagnosis. Six *CPA4 *single-nucleotide polymorphisms were genotyped, and evaluated for their relation to prostate cancer. We also evaluated whether CPA4 variants influence risk of disease among men diagnosed at an earlier age (< 66 years).

**Results:**

The nonsynonymous coding SNP (rs2171492, Cys303Gly) in *CPA4 *was associated with an increased risk of aggressive prostate cancer among younger patients (< 66 years). Specifically, men carrying the TT genotype had an approximately two-fold increased risk for being diagnosed with intermediate-to-high risk disease (Odds Ratio = 1.83, p = 0.04). In the overall population (all ages) none of the *CPA4 *SNPs demonstrated a statistically significant association with prostate cancer.

**Conclusion:**

Coding variation in *CPA4 *may confer increased risk of intermediate-to-high risk prostate cancer among younger patients. Further work is needed to identify the functional aspects of this variation and understand its biological effects on prostate cancer. Such work may translate into more precise screening of higher risk individuals as well as guiding clinicians and patients toward earlier and more definitive treatment modalities in patients genetically identified as higher risk.

## Background

Prostate cancer is the most common malignancy among men living in the United States. In 2008, an estimated 186,320 new cases of prostate cancer will be diagnosed, and the disease is estimated to be responsible for 28,660 deaths.[1] Identifying which cases harbor potentially aggressive disease versus those that will follow a more indolent course is extremely important given the established stage migration in prostate cancer as well as the growing popularity of active surveillance and minimally invasive therapies. Such knowledge would allow clinicians to more appropriately counsel patients and direct more aggressive therapies to those most in need while optimizing quality of life in those patients who are at lower risk for disease aggressiveness.

Previous work has contributed greatly to our understanding of disease aggressiveness and our ability to predict clinical outcomes. [2-7] The most powerful predictor variables have been a function of biochemical findings (PSA level), low-magnification histologic architecture (Gleason Grade), and physical exam findings (clinical T-stage). Predictive instruments (nomograms) have occasionally been developed which include novel biochemical markers, but these have generally offered only modest improvements in predictive accuracies over previous nomograms and have had limited generalizability.

The recent growth in our understanding of the human genome.[8] provides an opportunity to further understand the genetic basis of prostate cancer aggressiveness. We previously undertook a genome-wide linkage study and detected linkage between genetic markers on chromosome 7q32 and Gleason Grade (p = 0.0007).[9] This finding suggests that the 7q32 region might harbor genes for prostate cancer aggressiveness. Within this 7q32 region is *CPA4 *(previously identified as *CPA3*), part of the carboxypeptidase gene family and a strong candidate for the putative prostate cancer aggressiveness gene.

Huang et al.[10] used mRNA differential display to identify genes induced by butyrate in androgen-independent prostate cancer cells (PC-3). They found that during differentiation and apoptosis CPA4 mRNA was highly up-regulated. Meanwhile, they confirmed that CPA4 expression was extremely low in normal prostate tissue by RT-PCR analysis. They concluded that CPA4 appeared to be a downstream gene in response to the hyperacetylating activity of histones. Because of its structural homology to other carboxypeptidases, CPA4 is thought to modulate the function of peptide hormones that play an essential role in the growth and/or differentiation of prostate epithelial cells.[10] Natural substrates of carboxypeptidases include kinins, enkephalin hexapeptides, anaphylatoxins, and creatinine kinase.[11] CPA4 is imprinted preferentially from the maternal allele, and is hypothesized to impact prostate cancer aggressiveness.[12] While the specific ligand for CPA4 is yet to be discovered, there is a known endogenous protein inhibitor, latexin.[13] In light of this strong biological rationale, we present here the first investigation of genetic variation in CPA4 and prostate cancer aggressiveness.

## Methods

The study was comprised of 1,012 men: 506 diagnosed with intermediate-to-high risk prostate cancer (D'Amico classification)[14] and 506 age-, ethnicity-, and hospital-matched controls. All patients were recruited from the major medical institutions of Cleveland, Ohio between 2001 and 2004. These cases are considered representative of men diagnosed with intermediate-to-high risk prostate cancer in the greater Cleveland region. Cases were defined as newly diagnosed prostate cancer with histologically-confirmed disease demonstrating any of the following: Gleason score ≥ 7; clinical stage ≥ T2c; or PSA > 10 ng/ml at diagnosis. Cases were promptly contacted following diagnosis for inclusion in the study (median time from diagnosis to recruitment was 4.7 months). The restriction of cases to men with features of intermediate-risk and high-risk disease was performed in effort to focus on the most clinically relevant prostate cancers. To further focus on such cancer, we also stratified our analyses by age of onset (using 62 years as a cutpoint), and by whether the cases had prostate cancer with Gleason ≤ 3+4 versus cases with Gleason ≥ 4+3.

Controls were chosen among those who underwent standard annual medical examinations at the collaborating institutions. Controls had no diagnosis of prostate or any other non-skin cancers. All controls received a PSA test and were referred for urologic consultation if their PSA was ≥ 4 ng/mL. Controls were frequency matched to cases by age (within 5 years), ethnicity, and medical institution. Institutional review board approval was obtained from the participating institutions and all participants gave informed consent. A more detailed description of this study population has previously been reported.[15]

We used HapMap data http://www.hapmap.org/ and HaploView to select haplotype tagging SNPs to evaluate *CPA4*. We first selected single-nucleotide polymorphisms across the *CPA4 *gene which had minor allele frequencies (MAF) > 0.05. Seven SNPs in the Caucasian HapMap population (release #16) met this criterion. We eliminated one synonymous SNP (rs2306848) from our analysis due to difficulty in designing primers for the genotyping assay, but replaced it with a highly correlated tag SNP rs1488009 tag SNP (r^2 ^= 1). One of the SNPs included in our analysis (rs2171492) is a nonsynonymous coding SNP (Cys303Gly). Table 1 lists the six SNPs used for genetic analysis. The distribution of MAFs were relatively similar among Caucasian and African American populations. The correlation (D' and r^2^) between these SNPs are presented [see Additional file 1]. Note that the second through sixth SNPs cover a large haplotype block in European Americans that spans much of *CYPA4*.

**Table 1:** Six *CPA4 *SNPs evaluated for association with aggressive prostate cancer

				Minor Allele Frequency*	
SNPs	Position^†^	Location	Allele Change	African Americans	Caucasians
rs901799	129718638	5'	C > A	0.13	0.15
rs3807344	129721455	intron	A > G	0.17	0.11
rs1569132	129725280	intron	A > G	0.39	0.43
rs1038628	129735249	intron	G > T	0.20	0.40
rs2171492	129737976	exon^§^	A > G	0.35	0.36
rs1488009	129745334	intron	G > T	0.23	0.38

*minor allele frequency among controls.

^†^SNP position based on dbSNP build 129.

^§^rs2171492 = Cys303Gly

Genotyping was done by the 5' nuclease Taqman allelic discrimination assay using the manufacturer's predesigned primer/probe sets, and assays were read on a 7900 HT Sequence Detection System (Applied Biosystems, Foster City, CA). All assays were performed by individuals blinded to case-control status of the samples. The SNPs were all in Hardy-Weinberg equilibrium among cases and controls of each ethnic group (p > 0.05). For quality control, 2% replicate samples were included. The concordance rate for replicate samples was 100%.

Odds ratios (ORs) and 95% confidence intervals (CIs) were estimated by unconditional logistic regression to examine the association between the CPA4 variants and prostate cancer risk, adjusting for the matching factors. Haplotype frequencies were estimated by the expectation-maximization algorithm using the tagSNP software. Common haplotypes were defined as having a frequency ≥ 5%. Haplotype dosage (i.e. an estimate of the number of copies of haplotype h) for each individual and each haplotype, h, was computed using that individual's genotype data and haplotype frequency estimates obtained from the E-M algorithm. All reported *P*-values are two-sided, and all analyses were undertaken with SAS software (version 9.1; SAS Institute, Inc, Cary, North Carolina).

## Results

Baseline demographic characteristics for the 506 cases and 506 controls are presented in Table 2. The mean age was 65.7 years among cases and 65.5 years among controls. 82% of the study population was Caucasian and 18% was African-American. A family history of prostate cancer (defined as two or more 1^st ^degree relatives or one 1^st ^degree relative and two or more 2^nd ^degree relatives) was more commonly reported among cases than controls (6% vs. 1%, respectively). Eighty-four percent of cases had a Gleason score of ≥ 7 and 36% had cT-stage ≥ T2. Two hundred thirty-eight cases were diagnosed at age < 66 years; 40 (16.8%) of these patients were African-American. Younger onset cases are of interest here, as these are more likely to have a genetic basis to their disease.

**Table 2:** Characteristics of prostate cancer cases and controls included in association study

	Cases (N = 506)	Controls (N = 506)
Age (mean ± SD)	65.7 ± 8.2	65.6 ± 8.3
		
Ethnicity n (%)		
African-American	89 (18)	89 (18)
Caucasian	417 (82)	417 (82)
		
Institution n (%)		
Cleveland Clinic Foundation	407 (80)	407 (80)
University Hospitals, Cleveland	99 (20)	99 (20)
		
Family history of prostate cancer n (%)		
Yes*	31 (6)	7 (1)
No	475 (94)	499 (99)
		
Tumor Stage† n (%)		
T1	305 (60)	NA
T2	145 (29)	NA
T3	30 (6)	NA
		
Gleason Score n (%)		
5	3 (1)	NA
6	79 (16)	NA
7	314 (62)	NA
8	67 (13)	NA
9	39 (8)	NA
10	4 (1)	NA
		
PSA ng/ml (mean ± SD)	14.1 ± 27.4	1.7 ± 1.7

*1st degree relatives ≥ 2 or one 1st degree and 2nd degree relatives ≥ 2.

^†^Numbers do not add to 100% due to missing data

Among younger men the rs2171492 nonsynonymous coding SNP in *CPA4 *was associated with an increased risk of intermediate-risk and high-risk prostate cancer (Table 3). When restricting our analyses to men either diagnosed at age < 66 years (for cases) or enrolled into the study at age < 66 (for controls) carrying the TT genotype for the rs2171492 SNP versus the GG genotype gave a nearly two-fold increased risk for aggressive disease (odds ratio = 1.83, 95% confidence interval 1.02–3.36, p = 0.04). This association among younger men was strengthened when further restricting our analyses to cases with the most advanced disease (Gleason ≥ 4+3): men with the TT genotype for the rs2171492 SNP versus the GG genotype gave an odds ratio = 2.48 (95% confidence interval 1.14–5.40, p = 0.02). When the analysis was applied the overall study population (all ages), we did not observe any statistically significant associations between *CPA4 *and prostate cancer (Table 4).

**Table 3:** Association between *CPA4 *variants and advanced prostate cancer, stratified by age at diagnosis (using 66 years as cutpoint)

		Age < 66 years				Age ≥ 66 years			
		
SNP	Genotype	Cases	Controls	Odds Ratio (95% CI)*	p-value	Controls	Cases	Odds Ratio (95% CI)*	p-value
rs901799	CC	178 (74.8)	173 (72.7)	1		199 (74.3)	185 (69.0)	1	
	CA	56 (23.5)	61 (25.6)	0.89 (0.59–1.36)	0.60	60 (22.4)	75 (28.0)	1.35 (0.91–2.00)	0.14
	AA	4 (1.7)	4 (1.7)	0.98 (0.24–4.00)	0.98	9 (3.4)	8 (3.2)	0.96 (0.36–2.54)	0.93
									
rs3807344	AA	109 (45.8)	90 (37.8)	1		111 (41.4)	104 (38.8)	1	
	AG	98 (41.2)	120 (50.4)	0.67 (0.46–0.99)	0.05	124 (46.3)	123 (45.9)	1.06 (0.73–1.53)	0.76
	GG	31 (13.0)	28 (11.8)	0.91 (0.51–1.64)	0.76	33 (12.3)	41 (15.3)	1.33 (0.78–2.26)	0.30
									
rs1569132	AA	85 (35.7)	82 (34.5)	1		83 (31.0)	87 (32.5)	1	
	AG	98 (41.2)	121 (50.8)	0.78 (0.52–1.17)	0.23	131 (48.9)	136 (50.8)	0.99 (0.67–1.46)	0.96
	GG	55 (23.1)	35 (14.7)	1.51 (0.90–2.55)	0.12	54 (20.2)	45 (16.8)	0.80 (0.48–1.31)	0.37
									
rs1038628	GG	106 (44.5)	107 (45.0)	1		105 (39.2)	103 (38.4)	1	
	GT	91 (38.2)	102 (42.9)	0.91 (0.61–1.35)	0.62	119 (44.4)	129 (48.1)	1.11 (0.76–1.61)	0.60
	TT	41 (17.2)	29 (12.2)	1.44 (0.82–2.53)	0.20	44 (16.4)	36 (13.4)	0.83 (0.49–1.41)	0.50
									
rs2171492	GG	107 (45.0)	108 (45.4)	1		104 (38.8)	109 (40.7)	1	
	GT	90 (37.8)	107 (45.0)	0.86 (0.58–1.27)	0.44	126 (47.0)	126 (47.0)	0.95 (0.66–1.38)	0.79
	TT	41 (17.2)	23 (9.7)	1.83 (1.02–3.36)	0.04	38 (14.2)	33 (12.3)	0.83 (0.48–1.42)	0.49
									
rs1488009	AA	186 (78.2)	190 (79.8)	1		197 (73.5)	216 (80.6)	1	
	AG	49 (20.6)	46 (19.3)	1.09 (0.69–1.72)	0.71	68 (25.4)	44 (16.4)	0.58 (0.38–0.90)	0.01
	GG	3 (1.3)	2 (0.8)	1.53 (0.25–9.31)	0.65	3 (1.1)	8 (3.0)	2.47 (0.64–9.46)	0.19

* From unconditional logistic regression model adjusted for age, ethnicity, and medical institution.

**Table 4:** Association between *CPA4 *variants and advanced prostate cancer

SNP	Genotype	Cases, n (%)	Controls, n (%)	Odds Ratio (95% CI)*	p-value
rs901799	CC	363 (71.7)	372 (73.5)	1	
	CA	131 (2.6)	121(23.9)	1.11 (0.83 – 1.48)	0.46
	AA	12 (2.4)	13 (2.6)	0.95 (0.43 – 2.1)	0.90
					
rs3807344	AA	213 (42.1)	201 (39.7)	1	
	AG	221 (43.7)	244 (48.2)	0.85 (0.66 – 1.11)	0.25
	GG	72 (14.2)	61 (12.1)	1.11 (0.75 – 1.65)	0.59
					
rs1569132	AA	172 (34.0)	165 (32.6)	1	
	AG	234 (46.2)	252 (49.8)	0.89 (0.67 – 1.18)	0.41
	GG	100 (19.8)	89 (17.6)	1.08 (0.75 – 1.54)	0.68
					
rs1038628	GG	209 (41.3)	212 (41.9)	1	
	GT	220 (43.5)	221 (43.7)	1.01 (0.77 – 1.33)	0.94
	TT	77 (15.2)	73 (14.4)	1.07 (0.73 – 1.57)	0.78
					
rs2171492	GG	216 (42.7)	212 (41.9)	1	
	GT	216 (42.7)	233 (46.0)	0.91 (0.7 – 1.19)	0.49
	TT	74 (14.6)	61 (12.1)	1.19 (0.8 – 1.77)	0.38
					
rs1488009	AA	402 (79.4)	387(76.5)	1	
	AG	93 (18.4)	114 (22.5)	0.78 (0.57 – 1.07)	0.12
	GG	11 (2.2)	5 (0.01)	2.12 (0.73 – 6.16)	0.17

* From unconditional logistic regression model, adjusted for age, ethnicity, and medical institution.

To further investigate the association, we evaluated whether haplotypes defined by the SNPs across *CPA4 *were associated with disease. Only one common haplotype with a 35.1% and 15.9% frequency among Caucasian and African American men, respectively, contained the rs2171492 T allele. Men carrying two copies of this haplotype versus no copy was weakly associated with intermediate-risk and high-risk prostate cancer among men < 66 years of age (odds ratio = 1.82, 95% confidence interval = 0.98–3.36, p = 0.06). This association was slightly weaker than the result presented above for the rs2171492 TT genotype alone, suggesting that this SNP itself or another variant in linkage disequilibrium with the SNP but not on the haplotype might explain the association. Note also that restricting our analyses to Caucasians did not materially change our results (not shown).

## Discussion

This evaluation of genetic variants across *CPA4 *detected a positive association between the nonsynonymous coding SNP (Cys303Gly) rs2171492 and high risk prostate cancer among men with an earlier age of disease onset. Moreover, the SNP-level association was slightly stronger than that observed for the common haplotype carrying this SNP, suggesting that the SNP itself or another variant in linkage disequilibrium with the SNP but not on the haplotype may directly impact development of the more aggressive forms prostate cancer. If this finding is replicated by others, it would allow clinicians to focus treatment efforts in these higher risk patients. The potential for a direct relationship between a SNP and aggressive prostate cancer provides impetus to evaluate the functionality of this SNP.

*CPA4 *localizes to chromosome 7q32, a region that has been previously identified as a candidate region for prostate cancer aggressiveness.[9] CPA4 is upregulated by sodium butyrate (a known histone deacetylase inhibitor, HDACI) in treated cells from the androgen-independent prostate cancer cell line PC-3.[10] HDACIs have been studied extensively as potential chemotherapeutic and chemopreventive agents for various cancers including prostate cancer.[16,17] They have been shown to modify the expression of different genes, inhibit the cell cycle, and induce apoptosis in several cell lines.[18] Therefore, there is a potential biological basis to suspect that the rs2171492 SNP may alter the function of *CPA4 *with alterations in cell cycle regulation and potential carcinogenesis.

In light of the heterogeneous nature of prostate cancer[18], it might be easiest to detect genetic risk factors for this disease among earlier onset and more aggressive cases. Furthermore, it is precisely in these younger and more aggressive cases where early detection with definitive therapeutic intervention stands to yield the greatest impact on patients' lives. The identification of such genetic risk factors may translate into more precise screening of higher risk individuals and guide clinicians and patients toward earlier and more definitive treatment modalities in patients genetically identified as higher risk.

## Conclusion

Our study suggests that the rs2171492 nonsynonymous coding SNP in *CPA4 *confers an increased risk of high risk prostate cancer among younger patients. Additional research efforts are needed to confirm this finding, and if confirmed identify the functional aspects of this variant and understand their biological effects on prostate cancer.

## Abbreviations

PSA: (prostate-specific antigen); CPA4: (carboxypeptidase 4); SNPs: (single nucleotide polymorphisms); T-stage: (tumor stage); CPA3: (carboxypeptidase 3); OR: (odds ratio); CI: (confidence interval).

## Competing interests

The authors declare that they have no competing interests.

## Authors' contributions

PLR assembled and interpreted the data and drafted the manuscript. IC interpreted the data and assisted with drafting the manuscript. XL performed data analysis and interpretation and reviewed the manuscript. MC performed the genotyping assay. PRC provided intellectual content and reviewed the manuscript. GC was involved with the conception and design of the study. JSW was involved with the conception and design of the study, provided intellectual content and direction, drafted and reviewed the manuscript. All authors read and approved the final manuscript.

## Sources of funding

National Institutes of Health grants CA88164, CA112355, CA98683, and the California Urology Foundation.

## Pre-publication history

The pre-publication history for this paper can be accessed here:

http://www.biomedcentral.com/1471-2407/9/69/prepub

## Supplementary Material

Additional file 1 Pairwise correlation (D' and r2) between the *CPA4 *SNPs*. Pairwise correlation of the six SNPs use for *CPA4 *genetic analysis. Click here for file
