# Supplementary material for: Carboxypeptidase 4 gene variants and early-onset intermediate-to-high risk prostate cancer
Source: BMC Cancer. 2009 Feb 26;9:69. doi: 10.1186/1471-2407-9-69 (PMC2657151; doi:10.1186/1471-2407-9-69)
Supplement: Additional file 1 — Pairwise correlation (D' and r2) between the CPA4 SNPs*. Pairwise correlation of the six SNPs use for CPA4 genetic analysis. [file 1471-2407-9-69-S1.doc]

| **Supplemental Table 1**. Pairwise correlation (D' and r2) between the *CPA4* SNPs* | | | | | | |
| --- | --- | --- | --- | --- | --- | --- |
| African-Americans | | | | | | |
|  | rs901799 | rs3807344 | rs1569132 | rs1038628 | rs2171492 | rs1488009 |
| rs901799 |  | 0.01 | 0.57 | 0.21 | 0.43 | 0.37 |
| rs3807344 | 0.00 |  | 0.14 | 1.00 | 0.27 | 1.00 |
| rs1569132 | 0.07 | 0.00 |  | 0.66 | 0.49 | 0.65 |
| rs1038628 | 0.03 | 0.05 | 0.16 |  | 1.00 | 0.88 |
| rs2171492 | 0.02 | 0.03 | 0.09 | 0.13 |  | 0.37 |
| rs1488009 | 0.07 | 0.06 | 0.19 | 0.66 | 0.02 |  |
|  |  |  |  |  |  |  |
| Caucasians | | | | | | |
|  | rs901799 | rs3807344 | rs1569132 | rs1038628 | rs2171492 | rs1488009 |
| rs901799 |  | 0.10 | 0.23 | 0.28 | 0.22 | 0.10 |
| rs3807344 | 0.01 |  | 0.79 | 0.81 | 0.94 | 0.62 |
| rs1569132 | 0.01 | 0.10 |  | 0.95 | 0.96 | 0.89 |
| rs1038628 | 0.02 | 0.13 | 0.79 |  | 0.95 | 0.95 |
| rs2171492 | 0.01 | 0.06 | 0.40 | 0.34 |  | 0.94 |
| rs1488009 | 0.00 | 0.08 | 0.64 | 0.82 | 0.31 |  |
| *Above the diagonal are D' values between SNPs, and below the diagonal are r^2^ values between SNPs. | | | | | |  |
